# Rainbow: a tool for large-scale whole-genome sequencing data analysis using cloud computing

**DOI:** 10.1186/1471-2164-14-425

**Published:** 2013-06-27

**Authors:** Shanrong Zhao, Kurt Prenger, Lance Smith, Thomas Messina, Hongtao Fan, Edward Jaeger, Susan Stephens

**Affiliations:** 1 Systems Pharmacology and Biomarkers, Janssen Research & Development, LLC, 3210 Merryfield Row, San Diego, CA, 92121, USA; 2 Application Services Research & Development, Johnson & Johnson Services, Inc., New Brunswick, NJ, USA; 3 Translational Informatics IT, Janssen Research & Development, LLC, 3210 Merryfield Row, San Diego, CA, 92121, USA

**Keywords:** Cloud computing, Whole genome sequencing, Single nucleotide polymorphism, SNP, Next generation sequencing, Software

## Abstract

**Background:**

Technical improvements have decreased sequencing costs and, as a result, the size and number of genomic datasets have increased rapidly. Because of the lower cost, large amounts of sequence data are now being produced by small to midsize research groups. Crossbow is a software tool that can detect single nucleotide polymorphisms (SNPs) in whole-genome sequencing (WGS) data from a single subject; however, Crossbow has a number of limitations when applied to multiple subjects from large-scale WGS projects. The data storage and CPU resources that are required for large-scale whole genome sequencing data analyses are too large for many core facilities and individual laboratories to provide. To help meet these challenges, we have developed Rainbow, a cloud-based software package that can assist in the automation of large-scale WGS data analyses.

**Results:**

Here, we evaluated the performance of Rainbow by analyzing 44 different whole-genome-sequenced subjects. Rainbow has the capacity to process genomic data from more than 500 subjects in two weeks using cloud computing provided by the Amazon Web Service. The time includes the import and export of the data using Amazon Import/Export service. The average cost of processing a single sample in the cloud was less than 120 US dollars. Compared with Crossbow, the main improvements incorporated into Rainbow include the ability: (1) to handle BAM as well as FASTQ input files; (2) to split large sequence files for better load balance downstream; (3) to log the running metrics in data processing and monitoring multiple Amazon Elastic Compute Cloud (EC2) instances; and (4) to merge SOAPsnp outputs for multiple individuals into a single file to facilitate downstream genome-wide association studies.

**Conclusions:**

Rainbow is a scalable, cost-effective, and open-source tool for large-scale WGS data analysis. For human WGS data sequenced by either the Illumina HiSeq 2000 or HiSeq 2500 platforms, Rainbow can be used straight out of the box. Rainbow is available for third-party implementation and use, and can be downloaded from http://s3.amazonaws.com/jnj_rainbow/index.html.

## Background

High-throughput next-generation sequencing (NGS) technologies from Illumina (San Diego, CA, USA), Life Technology (Carlsbad, CA, USA), and Roche/454 (Branford, CT, USA) have evolved rapidly and are reshaping the scope of genomics research [1-3]. Technical improvements have greatly decreased sequencing costs and, as a result, the size and number of datasets have increased dramatically. The lower costs mean that sequence data are being produced more often by small to midsize research groups. This trend is likely to continue as newer generation sequencing technologies keep driving costs down [4]. The increasing volume of data has enabled the rapid adoption of whole-genome sequencing (WGS) to enhance drug research and development, which has led to a significant increase in the need for computational methods and bioinformatics tools [5,6]. For example, deep sequencing (30–60× fold coverage) of the entire human genome on an Illumina’s HiSeq 2000 platform typically generates billions of 100-bp short reads, and the corresponding FASTQ files can be as large as 460 gigabytes (GBs). For a WGS project consisting of 50 subjects, 20 terabytes of disk space are required to store the raw reads alone. The data storage and CPU resources needed pose a huge practical challenge for data analyses in a local environment.

Fortunately, in recent years, cloud computing has emerged as a viable option to quickly and easily acquire the computational resources for large-scale data analyses [7-9]. Cloud computing offers network access to computational resources where CPU, memory, and storage are accessible in the form of virtual machines (VMs). Using these VMs eliminates the need to build or administer local infrastructure while addressing the challenges involved in the rapid deployment of computing environments for bioinformatics. In addition, cloud computing offers machines with different hardware and software specifications, including large-memory machines, fast-CPU machines, and abundant disk space. In addition, users can select and configure VMs to meet their different computational needs. More importantly, cloud computing can provide storage and computation at a far lower cost (both up-front costs and on-going costs) than resources that are often dedicated to specific projects. With the massive economies of scale, cloud-computing providers are continually driving costs down. This has led to considerable enthusiasm within the bioinformatics community for the use of cloud computing for NGS sequence analyses [10]. Several cloud-based bioinformatics applications and resources have been developed specifically to address the challenges of working with the very large volumes of data generated by NGS technology. Cloud-based bioinformatics software include CloudBurst [11], Crossbow [12,13], Myrna [14], and CloVR [15].

Our focus has been on identifying genetic variations from WGS data, mainly single nucleotide polymorphisms (SNPs). To do this, all the short reads are aligned to a human reference genome and then SNP calls are made. A few open-source applications are available for mapping large numbers of short reads to reference sequences, including Bowtie [16], SOAP [17], and BWA [18]. These tools were designed initially for implementation on a single compute node or on a local workstation, and generally require a long running time even with multiple threads, making them impractical for processing a large number of samples. However, when a software program is executed in a compute cluster in which many processors work in parallel, the calculations can be completed in significantly less time. We used Crossbow [12], which is a Hadoop-based [19] parallel pipeline for genomic analysis, to search for SNPs using cloud computing. Crossbow uses Bowtie [16] and SOAPsnp [20] for alignment and SNP calling, respectively. Crossbow harnesses cloud computing to efficiently and accurately align billions of reads and call SNPs in a few hours. Data from an over 35× coverage of a human genome can be analyzed by Crossbow in three hours for 85 US dollars using a 40-node, 320-core cluster hosted in the Amazon cloud. When Crossbow was applied to large WGS projects in which multiple subjects were sequenced, various limitations were observed. The focus of this paper is to describe the development of Rainbow to address these limitations, and to demonstrate its practical usage by analyzing the genomic sequencing data from a large number of subjects in the Amazon cloud.

## Implementation

### Gaps and challenges for large-scale WGS analysis using cloud computing

A major challenge with WGS analysis in the cloud is the process of transferring large data files. The raw sequence data generated by large-scale WGS studies are generally multiple terabytes (TB) in size. It is impractical to transfer this data to the Amazon cloud via a typical network connection. Amazon Import is an efficient service for uploading large volumes of data to the Amazon S3 (Simple Storage Service) platform [21]. Users can ship multiple hard drives containing their data to Amazon via FedEx. Amazon then copies the data directly to S3. This process usually takes two to three days. After the data are uploaded to S3, the large files still need to be transferred between the S3 platform and the Amazon’s EC2 (Elastic Cloud Compute) instance [22]; this remains a practical challenge that is yet to be resolved. Currently, Amazon does not offer a built-in command line tool to facilitate the high-throughput transfer of large files between S3 and an EC2 instance.

Crossbow uses the s3cmd command line tool to download data from S3 [23], but s3cmd cannot handle data files larger than 5 GB. A typical FASTQ file for WGS is a few hundred gigabytes, much larger than the limit for s3cmd. [Author’s note: at the time of writing, a new alpha version of s3cmd was released that addresses the 5 GB limit.] Therefore, FASTQ files have to be split into smaller files for Crossbow runs. When Crossbow is executed in a cluster, it loads sequence data in multiple files in parallel to multiple nodes. Thus, file splitting reduces the data transfer time when Crossbow is run in a cluster. We developed a data pre-processing Python script that automatically splits large FASTQ files into smaller files and generates the corresponding manifest files as inputs to Crossbow runs.

Multiple EC2 instances can be launched in Amazon to split raw sequence data files in parallel. An EC2 instance might fail, crash, hang, or run away, which is the second challenge for Rainbow, namely, managing and monitoring multiple EC2 instances in the Amazon cloud. For example, when 100 EC2 instances are launched in the cloud, it is not practical to manually monitor this number of remote instances by logging into them one by one. To be useful, Rainbow should be able to monitor and detect some common hardware and network failures, and respond accordingly. To manage large-scale WGS data analysis in the cloud it is necessary to keep track of the progress and status of the application’s execution, and collect and record runtime metrics such as processing times, transfer times, and file sizes. To address this issue, we developed a data pre-processing Python script to log the necessary information for monitoring and to store the logs in S3. The script also performs automated management to ensure no common, foreseeable errors occur; if they do, the script can handle them appropriately. Take data transfer as an example. Data transfer from S3 to EC2 can sometimes fail because of network congestion in the cloud. When this occurs, instead of immediately terminating the EC2 instance, the script waits for several minutes before re-fetching the data. Other similar examples of automated management to handle common problems encountered during development and testing have been built into the script.

The third challenge for Rainbow is the aggregation of SNPs from multiple samples in a WGS project. The outputs from SOAPsnp for the sequences from a single subject are chromosome-based plain text files in which each SNP is annotated in detail. Unfortunately, the SOAPsnp output is not in a standard format, making it difficult for other genome-wide association studies (GWAS) tools such as Plink [24] to use the data. Identification of SNPs is one of the first steps in a WGS project. Other tools are used in downstream analyses to understand the significance of the identified SNPs. To address this problem, we developed a Perl script that can aggregate all the SNPs from multiple samples and merge them into chromosome-based genotype files, thereby allowing the files to be used as inputs to other GWAS tools.

The fourth and final challenge for Rainbow is the delivery of data from sequence providers. No standard has been set for providers, so data can be delivered in a variety of different formats. Raw sequence data are usually delivered as FASTQ files by shipping multiple hard drives to the customers. However, sequence providers might run their analysis pipelines automatically after sequencing and deliver BAM files to the customers. (BAM is a binary version of a SAM (Sequence Alignment Map) file; SAM is a tab-delimited text file that contains sequence alignment data.) This reduces the number of multiple hard drives that are required for the data from large sequencing projects because a BAM file is roughly one-third the size of the corresponding FASTQ file. In a BAM file, all the raw reads have been aligned, but customers may want to redo the mapping using a different alignment program or a different reference genome (for example, the hg19 version of the human assembly instead of the hg18 version). When raw data are stored in the BAM format, the raw sequence reads first have to be extracted using Picard [25], and then re-aligned with Bowtie [16] or another alignment tool. Rainbow can use both BAM and FASTQ files as input and extracts sequence reads from BAM files on the fly.

### Cloud as the execution environment for Rainbow

Many CPU hours are required to align raw reads in large datasets to a reference genome. For a subject with 60× sequencing coverage, it takes about two weeks to map 2 billion reads using Bowtie [16] on a local Linux machine without parallel processing. On a single machine, it would take about three years to align all the reads in a WGS project consisting of 50 subjects. In principle, it is possible to set up a local high-performance computing cluster to meet the computational and storage challenges of large-scale WGS data analysis; however, this option is not always available. Furthermore, this option cannot be scaled up or down quickly to meet the needs of different sequencing projects. Cloud service providers give customers on-demand access to a wide range of cloud infrastructure services irrespective of the size of the data, and charge only for the resources that are actually used. Cloud service providers offer virtually unlimited storage and CPU resources, and provide a computational environment that is ideally suited to large-scale WGS data analyses.

We chose to build Rainbow using the Amazon Web Services as the cloud provider for the following reasons: (1) S3 centralizes data storage, including inputs, intermediate results, and outputs from every computational step in Rainbow, and stores the data permanently. A variety of tools are available to access the data stored in S3, such as S3Fox, a web browser plug-in [26], and s3cmd, a command line tool [23]. S3 provides virtually unlimited storage space for cloud computing, and data in S3 are stored as multiple copies. Multiple data copies and redundancy guarantee the safety of data in Amazon’s cloud. The objects in S3 seem to belong logically to the same folder, but they are not necessarily on the same physical device (a hard drive or a file system). All objects in S3 have unique identifiers and can be fetched in parallel without input/output (I/O) congestion. This parallelism is critical when multiple instances or clusters are uploaded to or downloaded simultaneously to files in S3, and EC2 instances or clusters requested by the user can be released after the computational tasks are complete, and the user no longer needs to pay for those resources.

### Manifest file and name convention makes the use of Rainbow easy

To support the centralization of all data files and to manage different types of files in S3 in an automated fashion, we used a master manifest file and naming convention. For a large WGS project, the manifest file is the only file that a user needs to prepare to run Rainbow. A master manifest file is a plain text file used to describe all subjects in a WGS project. Each subject has a corresponding entry in the manifest file. The format for each record is:

Test1 *   s3://test.bucket/test1.bam s3://test.results/*

Test2 *   s3://test.bucket/test2_1.fastq; s3://test.bucket/test2_2.fastq s3://test.results/*

Each entry consists of three fields separated by spaces or tabs: (1) a unique identifier; (2) locations of the raw reads in S3; and (3) an output folder in S3. The raw reads can be in either BAM or FASTQ files. The naming convention that we have used, together with the unique identifier for each genome, control how all the intermediate and result files are named, where they are stored, and how they are logically organized in S3. Each individual step in the Rainbow workflow uses this single manifest file as input, thus guaranteeing that all files are named and stored consistently. This process ensures that naming conflicts or overwriting of any file associated with another subject is avoided.

### Merging SNP outputs for multiple subjects

Individual SNP records generated using SOAPsnp are in SOAPsnp output format, namely, one SNP per line with several tab-separated fields per SNP. The fields include SNP coordinate information, subject genotype, quality score of subject genotype, the best base and the number of uniquely aligned reads corroborating the best base, and the second best base and the count of all aligned reads corroborating the second best base. For each subject, the SNPs are organized into one gzipped result file per chromosome, and the SNP records are sorted based on their coordinates on the chromosome. We developed a merge-sort-based algorithm to merge the SNPs chromosome by chromosome. When merging SNPs from multiple subjects, we wanted to ensure that only high-quality SNPs were retained for downstream analysis. The criteria that we used to define a quality SNP were: (1) the “quality score of subject genotype” attribute is greater than 13, giving at least 95% confidence that the genotype is correct. The number 13 was calculated using the formula − 10 * *log*(1 − 95*%*); and (2) at least two uniquely aligned reads corroborate both the best and the second best bases. During the SNP merging process, there is no need to load all the SNPs into memory at once. As a result, our algorithm has a very small memory footprint, and can merge SNPs from a very large number of subjects. The merged genotypes are stored as plain text files with one row corresponding to one SNP marker, and with each column corresponding to one sample.

## Results

### Description of Rainbow

**Figure 1:**
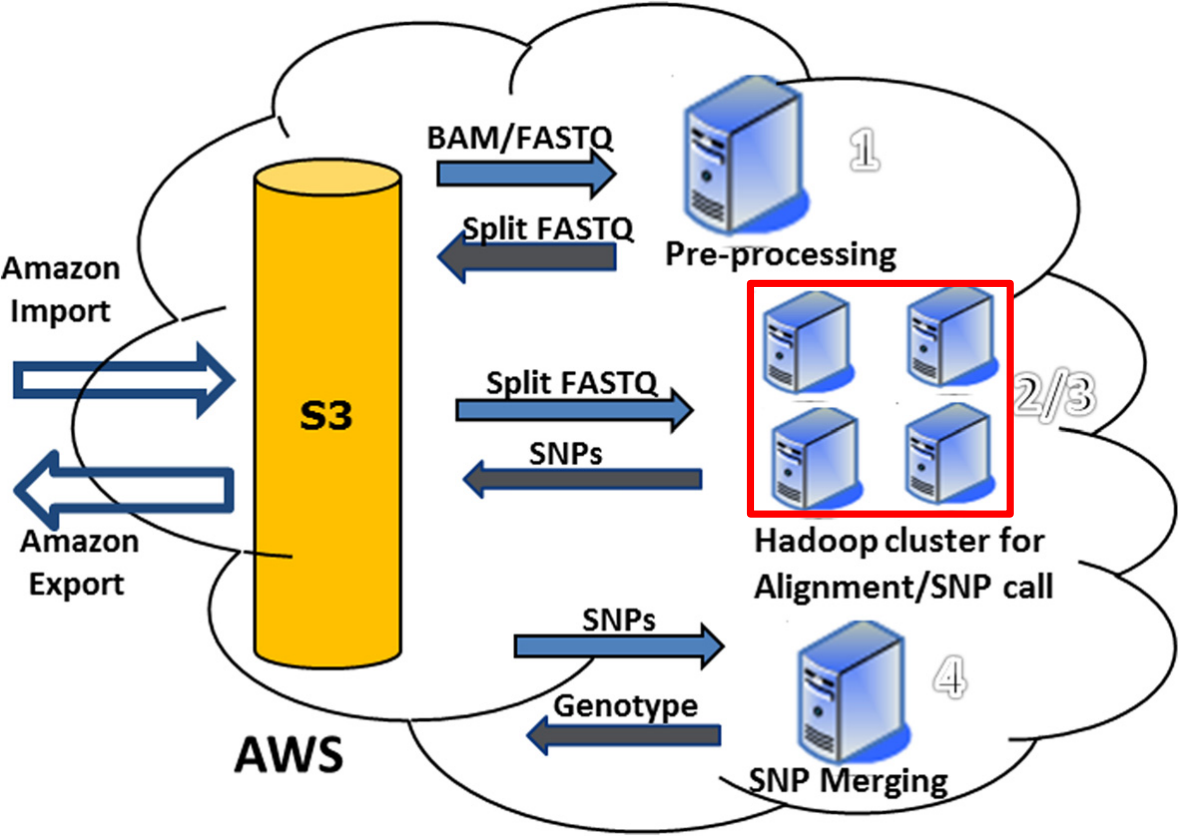
**The Rainbow pipeline.** S3 centralizes data storage, including inputs, intermediate results, and outputs. A typical scenario to run Rainbow to analyze large-scale WGS data is import → execute → export. Alignment and SNP call are performed by Crossbow in a cluster with multiple nodes. AWS, Amazon Web Service.

The workflow of Rainbow is shown in Figure 1. A user first ships multiple hard drives to Amazon via FedEx, and Amazon copies the data to S3 directly. This process typically takes two to three days. After the BAM or FASTQ files have been uploaded to S3, they can be processed in parallel by launching multiple EC2 instances or clusters in the cloud. When the analysis is complete, the results can be downloaded directly or exported back via Amazon Export, which takes an additional two to three days.

**Figure 2:**
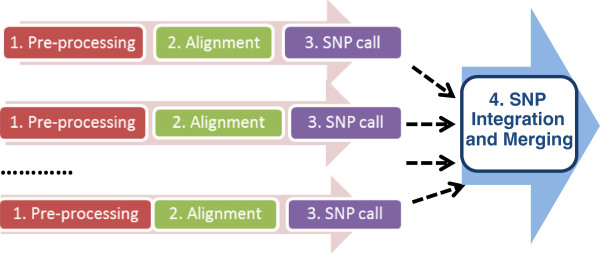
**The four main steps in WGS data analysis using Rainbow.** Steps 1, 2, and 3 can be executed in parallel in the cloud by launching multiple instances or clusters. Crossbow performs both the alignment and the SNP call.

There are four major steps in WGS data analysis (Figure 2). Step 1, the data pre-processing step, automates (1) the extraction of raw reads from BAM files; (2) the splitting of large FASTQ files into smaller files; and (3) the generation of manifest files as inputs to Crossbow. In step 1, some user data are passed to the EC2 instance through the Cloud-init mechanism [27]. This user data is an executable shell script that is responsible for downloading the pre-processing Python code from S3. The Python script is responsible for software installation, system configuration, fetching data from S3, extracting raw reads, splitting files, and uploading all the results to S3. Steps 2 and 3 are performed by Crossbow and are responsible for mapping reads to the reference sequence and for SNP calling. A Perl script is used to parse a master manifest file, prepare the Crossbow command line, and launch the Crossbow run in the cloud for each subject. Step 4 uses a Perl script that was developed to consolidate the SNPs for all samples.

### A practical test run

We applied Rainbow to analyze the 44 subjects listed in Table 1. All 44 subjects were pair-end sequenced on Illumina HiSeq 2000 platforms. The estimated insert size was approximately 300 bp. Each subject generated 1.1–2 billion 100-bp short reads. The largest BAM file was 190 GB, and the corresponding FASTQ files were 460 GB (2 × 230 GB). Sequencing coverage ranged from 30 to 60×. All the raw data were in BAM format and delivered to us on four 2 TB hard drives. We shipped the hard drives to Amazon’s Import Service and loaded the data files to S3. Then, we launched 44 m1 large instances in the Amazon cloud to pre-process the raw data in parallel and to upload all split files to S3. After data pre-processing, 44 clusters were launched in parallel in the cloud to align the reads and make SNP calls. Finally, the SNPs from all 44 subjects were merged and a master chromosome-based genotype file was generated for further analysis.

**Table 1:** Description of the WGS data for 44 subjects and a summary of the detected SNPs

**Sample**	**BAM File (GB)**	**FASTQ (GB)**	**#Reads(Millions)**	**Homo_SNPs**^**a**^	**Hetero_SNPs**^**b**^	**Het2_SNPs**^**c**^	**Total_SNPs**
SG1226	150	427	1900	1503239	2507716	2105	4013060
SG1227	84	269	1220	1508162	2452631	1768	3962561
SG1229	115	283	1280	1548421	2242797	1480	3792698
SG1230	111	301	1362	1494063	2581379	1906	4077348
SG1231	128	388	1740	1481840	2626852	2138	4120830
SG1232	97	258	1162	1497747	2729469	2468	4229684
SG1233	72	244	1204	1707533	2145142	1710	3854385
SG1234	103	333	1496	1550433	2790889	3233	4344555
SG1235	89	283	1258	1479108	2370792	1465	3851365
SG1236	96	311	1382	1490258	2752238	2800	4245296
SG1237	115	346	1552	1520718	2715088	2983	4238789
SG1238	102	322	1432	1504343	2327337	1457	3833137
SG1239	93	260	1170	1463430	2517434	1596	3982460
SG1240	91	251	1122	1472236	2439010	1599	3912845
SG1241	89	251	1120	1477814	2477763	1617	3957194
SG1242	97	300	1360	1501310	2470358	1856	3973524
SG1243	93	305	1356	1476636	2467669	1646	3945951
SG1244	95	269	1202	1477605	2462121	1613	3941339
SG1245	98	277	1244	1504849	2401576	1716	3908141
SG1246	97	269	1202	1496063	2474375	1747	3972185
SG1248	149	363	1632	1461303	2493556	1876	3956735
SG1249	126	382	1702	1484862	2502888	2005	3989755
SG1250	141	418	1860	1491431	2556946	2249	4050626
SG1251	144	418	1860	1507550	2516188	2161	4025899
SG1252	146	427	1918	1470888	2633297	2108	4106293
SG1253	142	432	1940	1492478	2594146	2158	4088782
SG1254	127	374	1682	1527922	2504784	1971	4034677
SG1255	138	392	1760	1472663	2622476	2130	4097269
SG1256	143	420	1872	1470631	2666175	2235	4139041
SG1257	122	381	1712	1481487	2555804	2118	4039409
SG1258	112	284	1278	1608618	2267405	1681	3877704
SG1259	124	330	1492	1463934	2661886	1991	4127811
SG1260	133	307	1382	1523274	2414508	1616	3939398
SG1263	112	349	1552	1470360	2653711	2075	4126146
SG1264	122	376	1690	1486174	2631507	2034	4119715
SG1265	101	307	1378	1489230	2495349	1800	3986379
SG1266	118	352	1576	1479540	2552931	2014	4034485
SG1267	99	310	1382	1486385	2490343	1874	3978602
SG1268	105	334	1488	1473782	3225627	2422	4701831
SG1269	114	358	1608	1512683	2477654	2043	3992380
SG1270	108	298	1340	1557762	2692982	2656	4253400
SG1271	127	388	1742	1516008	2596244	2149	4114401
SG1272	87	256	1134	1406043	2820949	1743	4228735
SG1273	183	461	1950	1488525	2393320	2188	3884033

^a^Homo_SNPs are SNPs where both alleles are the same but different from the reference.

^b^Hetero_SNPs are SNPs where one allele is the same as the reference and the other allele is different.

^c^Het2_SNPs are SNPs where both alleles are different from the reference, and different from each other.

The total cost of shipping the four 2 TB hard drives was 150 US dollars per 2 TB hard drive including (a) 30 US dollars for FedEx shipping, (b) a flat 80 US dollars charge per device, and (c) 42 US dollars for the data loading fee. The additional charge for data loading was 2.49 US dollars per data-loading-hour, and the actual cost is dependent upon the I/O speed of the storage device and the data size. It took one business day to ship the hard drives to Amazon by FedEx, and Amazon started to upload the data within 24 h of receiving the hard drives.

The running environments were as follows. For step #1, we chose the Amazon m1.large instance, which has two CPUs, 7.5 GB memory, and two 420 GB instance drives. For each instance, an extra 220 GB EBS (Elastic Block Storage) [28] volume was requested to store the raw BAM file. When running Picard [25], 6 GB memory was allocated to the Java Virtual Machine. Picard read the BAM file from EBS, and output pair-ended sequences into two FASTQ files (Fastq_1 and Fastq_2) stored in the two instance drives. When splitting files, Fastq_1 and Fastq_2 were processed in parallel because an m1.large instance has two CPUs, and Fastq_1 and Fastq_2 were logically in different instance drives. All requested resources (CPUs/storages) of the m1.large were used fully, which made it the optimal choice. For steps 2 and 3, each compute cluster had 40 c1.xlarge nodes as recommended by the Crossbow developers. Each c1.xlarge node has eight CPUs, 7 GB memory, and 1690 GB instance storage.

**Figure 3:**
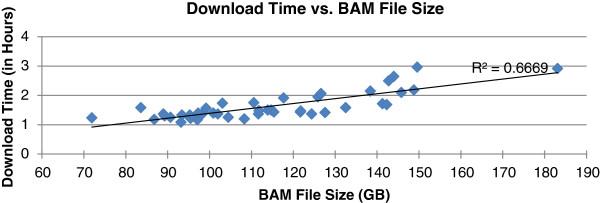
Download time from S3 to EC2 instance in the cloud versus the BAM file size.

**Figure 4:**
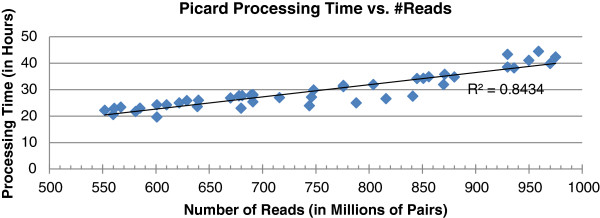
Running time of Picard versus the number of sequence reads.

**Figure 5:**
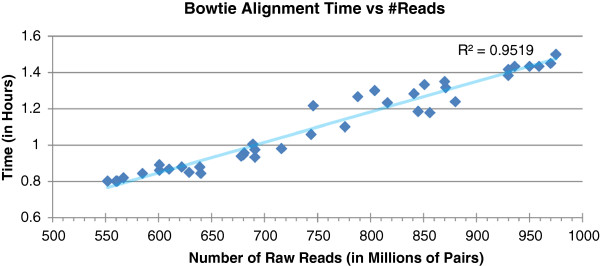
**Running time of Bowtie alignment versus the number of paired sequence reads.** The cluster consisted of 40 c1.xlarge instances.

**Figure 6:**
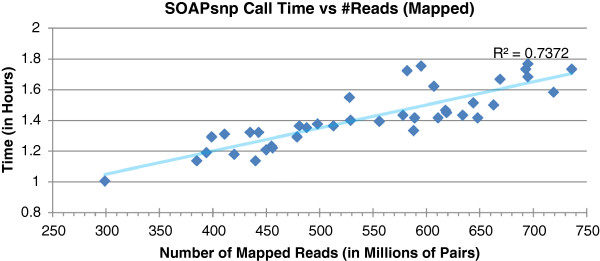
**Running time of SOAPsnp versus the number of paired mapped reads.** The cluster consisted of 40 c1.xlarge instances.

The performance of Rainbow is summarized in Figures 3, 4, 5, 6. The relationship between the download time and BAM file size is shown in Figure 3. Outliers of the trend line reflect fluctuations of network traffic. Downloading was performed using boto [29], an open source Python tool for easy connection to the Amazon Web Service. On average, a data transfer speed of 1.3 GB per min was achieved. Picard [25] comprises Java-based command-line utilities that manipulate BAM and SAM files. One of the utilities was used to extract raw sequence reads from BAM/SAM files. The more reads in a BAM file, the longer it takes to extract them, and the linear relationship between Picard processing time and the number of reads represents this (see Figure 4). It usually takes one to two days for Picard to complete a run on an m1.large node; it would not have been faster if another more powerful EC2 instance had been chosen. In addition, there is no way to run Picard in parallel to process a BAM file. The output of a Picard run is two large FASTQ files. It takes another eight to 16 h for FASTQ file splitting, compression, and uploading to S3. Step 1 will of course take much less time if the raw reads are in FASTQ files rather than in a BAM file.

Steps 2 and 3 are much more time consuming than step 1, but they can be completed in a much shorter time in a cluster with multiple nodes (see Figures 5 and 6). In a 320-CPU cluster, the alignment of billions of reads takes between 0.8 and 1.6 h, whereas, on a local resource, it could take up to 12 days. The linear relationship shown in Figure 5 is accurate because the sequence data blocks in the HDFS (Hadoop Distributed Filesystem) [30] were physically local to the nodes that processed them, which reduces virtual I/O delays. Crossbow runs rarely failed because the Hadoop-based cluster was built to run on commodity hardware, and Hadoop has built-in mechanisms for failover and disaster recovery. A Hadoop-based cluster not only reduces the running time by processing the data in parallel, but also significantly improves the robustness of applications. The SOAPsnp running time (see Figure 6) ranged from 1 to 1.8 h, which overall, was a little longer than step 2. The numbers of SNPs identified from the 44 subjects are listed in Table 1. On average, about 4 million SNPs were identified from a single subject. After combining the SNPs from all 44 subjects, roughly 15 million unique SNPs were obtained. It was very rare for both alleles to be different from the reference sequence, indeed, only a few thousand SNPs per subject fell into this category (Table 1).

On average, it cost less than 120 US dollars to analyze each subject, and the total cost for analyzing 44 subjects was around 5,800 US dollars, including data import. All EC2 instances and clusters are terminated immediately after the jobs on them finish, and the large amount of data in S3 can be deleted after data export to reduce continual charges. No upfront investment in infrastructure is required and there are virtually no additional administrative costs involved in using the Amazon Web Service. More important than the cost is the ability to scale Rainbow up or down so that the analyses are accomplished in a short time. Currently, we are working on a large whole-genome sequencing project in which 438 subjects are sequenced. Rainbow will be able to process the data from these 438 subjects in less than two weeks, including the physical data import and export with Amazon. Compared with the 30 years or so it would take to process this number of subjects sequentially on a local machine, the time savings of parallel processing enabled through Rainbow are obvious.

## Discussion

Rainbow was built on Crossbow, but the complexity of the Crossbow command line is hidden, which facilitates its use for large-scale WGS analysis in the cloud. Rainbow has been well tested, and has proven to be robust and scalable. The implementation is open-source based, and is available for third-party deployment and use. Illumina HiSeq 2000 and 2500 are currently the dominant sequencing platforms, and accordingly, the default parameters in Rainbow have been optimized and finely tuned for data generated by these platforms. User can tailor the parameters for other platforms, but this is rarely needed in practice. For human WGS data sequenced on an Illumina HiSeq 2000 or HiSeq 2500 platform, Rainbow can be used straight out of the box.

The low cost of whole genome sequencing has led to the rapid adoption of WGS for drug research and development. To understand the relationship between SNPs and disease better, and to obtain insights into the relation between SNPs and drug response, large-scale sequencing projects are continuously being initiated in academic institutes and drug companies [31,32]. These sequencing projects need high-performance computing capabilities for WGS data analyses. As we have shown, cloud computing drives down infrastructure costs both up-front and on an on-going basis, and offers operational advantages, such as setting up infrastructure in minutes rather than months, completing massive computational projects with a large number of resources quickly, and scaling the architecture up and down to provide the required computational resources.

Analyzing large datasets in the cloud is different from performing the same analysis in a local environment. By implementing and running Rainbow in the cloud, we have learned many valuable lessons. Here we summarize what we have learned while developing and testing Rainbow.

•Boot time should be taken into account when new resources are starting up. It is good practice to give cloud providers 10–15 min before attempting to use a newly requested resource.

•It is not trivial to move large datasets around in the cloud. Users should be prepared to handle network congestion or failures. When data transfers fail, it is advisable to wait for 5–10 min before retrying.

•Cloud providers typically offer a variety of compute instances to choose from. It is necessary to understand the bottleneck (CPU, I/O, or network) for the algorithm that is to be run, and choose the best option accordingly.

•When large amounts of data are moved between cloud resources, it is essential to ensure that they are in the same region or data center.

•It is difficult to debug workflows in the cloud without heavy logging.

## Conclusions

We have described the motivation and implementation of Rainbow for large-scale WGS data analyses in the cloud. Rainbow has the capacity to process more than 500 subjects in two weeks using the Amazon Web Service, including physical data import and export with Amazon. The average cost to process a single sample in the cloud was less than 120 US dollars. In essence, Rainbow is a wrapper of Crossbow, which can handle additional challenges in large-scale WGS data analyses. Compared with Crossbow, the main improvements of Rainbow include the ability (1) to handle BAM as well as FASTQ files as inputs, (2) to split large sequence files for better load balance downstream, (3) to log the running metrics in data processing and monitoring multiple EC2 instances, and (4) to merge SOAPsnp outputs for multiple subjects into a single file to facilitate downstream GWAS studies. Rainbow is scalable and easy to use.

## Availability and requirements

Rainbow is available for third-party use. Because it was built for use with Amazon Web Services, users need to first set up an Amazon account before launching Rainbow from a Linux machine. The source code for Rainbow is freely available for download; however, users need to pay Amazon to run analyses in the Amazon cloud. Detailed instructions on using Rainbow are available on the Rainbow website (http://s3.amazonaws.com/jnj_rainbow/index.html) [33].

## Abbreviations

VM: Virtual Machine; EC2: Elastic Compute Cloud; S3: Simple Storage Service; EBS: Elastic Block Storage; HDFS: Hadoop Distributed Filesystem; WGS: whole-genome sequencing; NGS: next generation sequencing; SAM: Sequence Alignment Map; BAM: binary version of SAM; SNP: single nucleotide polymorphism.

## Competing interests

The authors declare that they have no competing interests.

## Authors’ contributions

SZ designed, implemented, tested Rainbow, and wrote the manuscript. KP implemented pre-processing, performed tests, and collected results. LS helped with Amazon data import/export and performed tests. TM contributed to discussion in implementation. EJ offered computational resources for tests. KP, HF, and SS participated in writing the manuscript. All authors read and approved the final manuscript.
